# Stem Cell-Derived Exosomes Potential Therapeutic Roles in Cardiovascular Diseases

**DOI:** 10.3389/fcvm.2021.723236

**Published:** 2021-08-10

**Authors:** Selvaraj Jayaraman, Dhanavathy Gnanasampanthapandian, Johnson Rajasingh, Kanagaraj Palaniyandi

**Affiliations:** ^1^Department of Biochemistry, Saveetha Dental College and Hospitals, Saveetha Institute of Medical and Technical Sciences, Saveetha University, Chennai, India; ^2^Cancer Science Laboratory, Department of Biotechnology, School of Bioengineering, SRM Institute of Science and Technology, Chennai, India; ^3^Department of Bioscience Research & Medicine-Cardiology, The University of Tennessee Health Science Center, Memphis, TN, United States

**Keywords:** exosomes, cardiovascular therapy, miRNA, stem cells, cell therapy

## Abstract

Owing to myocardial abnormalities, cardiac ailments are considered to be the major cause of morbidity and mortality worldwide. According to a recent study, membranous vesicles that are produced naturally, termed as “exosomes”, have emerged as the potential candidate in the field of cardiac regenerative medicine. A wide spectrum of stem cells has also been investigated in the treatment of cardiovascular diseases (CVD). Exosomes obtained from the stem cells are found to be cardioprotective and offer great hope in the treatment of CVD. The basic nature of exosomes is to deal with the intracellular delivery of both proteins and nucleic acids. This activity of exosomes helps us to rely on them as the attractive pharmaceutical delivery agents. Most importantly, exosomes derived from microRNAs (miRNAs) hold great promise in assessing the risk of CVD, as they serve as notable biomarkers of the disease. Exosomes are small, less immunogenic, and lack toxicity. These nanovesicles harbor immense potential as a therapeutic entity and would provide fruitful benefits if consequential research were focused on their upbringing and development as a useful diagnostic and therapeutic tool in the field of medicine.

## Introduction

Cardiac problems and their related diseases are considered to be the major cause of morbidity and mortality around the World (1, 2). Reports suggest that 17.7 million population succumb to death every year due to the risk of cardiovascular diseases (CVDs), which claims 31% of the total deaths worldwide (1). According to the recent studies (3–5), naturally produced membranous vesicles have the potential capability of targeting the recipient cells specifically and delivering their bioactive constituents. Owing to this characteristic, these vesicles could emerge as a potential candidate in the field of regenerative medicine. This non-living entity but bioactive, termed as “exosomes”, shows promising preliminary results in mending a broken heart with the help of stem cells; thereby, proving their advantage over the use of other cell types. Exosomes are looked upon as a best tool with great emphasis on their nature of selectivity and the ease of uptake by target cells, thereby creating a platform bestowed with immense opportunities in cell and tissue-specific targeting (6–8).

**Figure 1:**
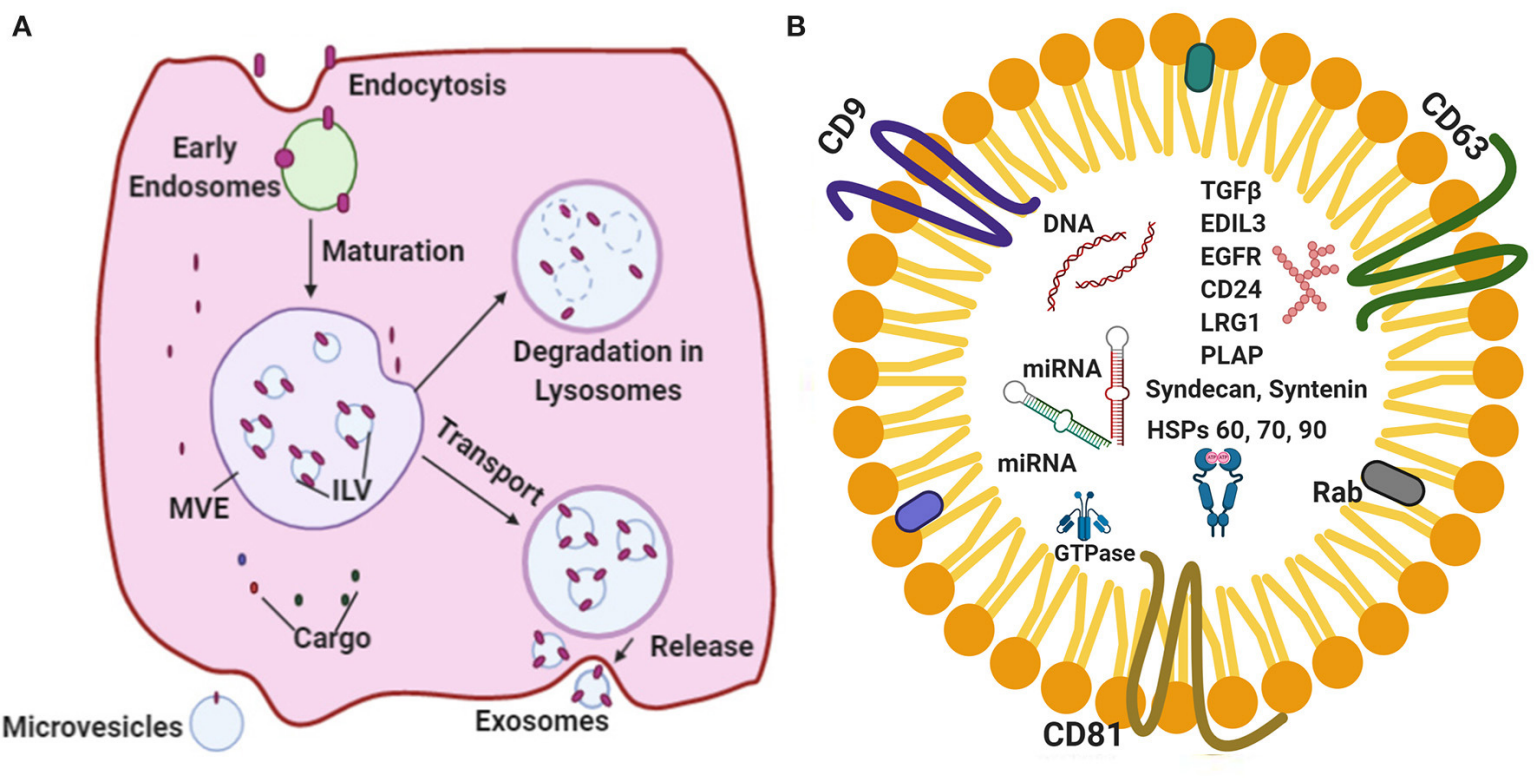
**(A)** A schematic representation of the major vital components of the endosomal pathway synthesis and release. **(B)** Represents standard exosomal -markers that have been reported to serve as biomarkers for the identification of certain diseases. Exosomal cargo composed of various proteins, mRNA, miRNA and DNA.

The cellular waste from the lining cells are known to be discarded by means of releasing secretory vesicles called as exosomes (9, 10). Recent studies (4, 11–14) have indicated that exosomes are escalated into crucial agents in cell–cell signaling and find application in normal physiology, such as myocardial angiogenesis, cardiac development, and the formation of vesicles during the maturation of reticulocytes. Exosomes bring about intracellular communication by way of particular modes, such as direct interaction by cell-to-cell contact, electrical, as well as long range signals and some chemical molecular interaction processed extracellularly (15–17). It has been established that one of the essential features required for the regulation of functions of the heart is the use of extracellular vesicles (EVs), precisely the exosomes (15, 18–20). The terminology “exosomes” were proposed by Johnstone et al. (21) while investigating the vesicle formation that are involved in the formation of reticulocytes. The exosome synthesis and release are diagrammatically represented (Figure 1A).

Exosomes are nanosized EVs that range from 30 to 150 nm in size with a floating density of 1.13–19 g/ml, as determined by using the ultracentrifugation technique on a linear sucrose density gradient (2–0.25 M of sucrose) (22–25). According to International Society for Extracellular Vesicles (ISEV) the membrane vesicles classified three categories apoptotic bodies (ABs), membrane vesicles (MVs) and exosomes. Exosomes possess unique characteristic features, such as a fluid lipid bilayer, surface proteins/receptors, mRNAs, microRNA (miRNA), specific set of proteins, transcription factors, and other substances (26, 27). Exosomes composed of various signaling molecules, miRNAs and nucleic acids are clearly depicted (Figure 1B). A previous study (15) indicated that the exosomal contents are highly regulated in the case of stress and disease conditions, which reflects the nature of the parent cell.

Exosomes belong to the unique subtype of membrane vesicles that are released from the vital endocytic compartment of live cells. The EVs develop from budding after the invagination of plasma membrane and are synthesized through the endosomal pathway (27, 28). The EVs with membranous structures are typically, otherwise, called as exosomes, microvesicles, microparticles, ectosomes, oncosomes, apoptotic bodies, and so on (29–31). The United States is known to be the major contributor and lead the World in terms of for the research on exosomes (32).

Various theories attribute to the mechanism of action of exosomes. First, exosomes possess proteomic potency that exerts a potent diverse effect on the immune responses from both humoral and cell-mediated components of the immune system (33–35). Second, exosomes play a pivotal role in disease-modulating capacity due to the transfer of miRNAs (36, 37). Third, exosomes are of endosomal origin predominantly and are composed of small (~30–140 nm) particles enclosed by a lipid bilayer (13). Finally, they are classified further as a subset of EVs that are secreted precisely by most of the cell types (16). The isolation and purification of exosomes are still underdeveloped (14, 16, 38, 39). The isolation of exosomes from raw biological fluids seems to be a herculean task as they are similar to the size of other components in the biological fluids, such as lipoprotein, chylomicrons, and macrovesicles, which overlap with that of the exosomes (17, 39, 40).

### Storage and Preservation of Exosomes

The suitable temperature to store exosomes has been found to be at −29°C (40). The anti-freezing agents are found to be helpful in preventing the formation of ice crystals inside the exosomes, increase their shelf-life, and help in storing them at −80°C or in liquid nitrogen (41–43). The entire protein content and representative functional analysis would be preserved in the exosomes if they are analyzed immediately after isolation (44). The isolation of exosomes was found to be easier from conditioned culture media and the entire process seems to be not complicated; however, different types of EVs are often co-isolated owing to the size overlap and the lack of cell-specific biomarkers (45). Many techniques have been devised for the purification of exosomes and these are also known to impact the yield, density, and function of recovered EVs (46–48). These techniques have been classified broadly into two subgroups: conventional methods and microfluidics-based methods (14, 49).

The conventional methods include size exclusion, chromatography, ultracentrifugation, immunoaffinity, ultrafiltration, and polymer-based precipitation (16, 50). These are well-established methods and are used widely, but known for lesser efficiency or reduced yield (51). In contrast, microfluidics-based method has gained momentum in the rapidly evolving isolation platforms as they possess many advantages, such as low sample consumption, increased sensitivity, easy to use, and tremendous speed when compared with conventional methods (52–54).

The purification of exosomes using the conventional method has been the most dependent mode over the past decade in research laboratories and clinics. The main principle underlying the method of isolation of exosomes is based on their physical property or their functions. As a result, they have been classified into three subgroups: density-based, size-based, and function-based isolation (16, 55).

In order to process small amount of fluid, microfluidics work on micron-sized channels (μL to pL) (52, 56). The microfluidic devices are fabricated with a specific polymer named polydimethylsiloxane (PDMS) (24, 57, 58). PDMS is known to be optically transparent and biocompatible (59). On the basis of application and separation approach required for the experiment, different components comprise the microfluidic device, such as microchannels, microvalves, connecting tubes, micropumps, and micromixers (60). Recent studies (52, 53, 61) have proved that microfluidics are capable of sorting exosomes with increased purity and sensitivity. At the same time, cutting down costs, reducing the amount of reagents utilized, and most importantly, bringing down the duration of time invested in the protocol drastically (62).

In general terms, microfluidics-based method has been classified broadly into two methods: active and passive (63). The former method relies on the exertion of external forces for its application, whereas the latter one is dependent mostly on the use of hydrodynamic and surface forces (64).

## Method of Exosome Uptake by Cells

The uptake of exosomes could be denoted as the process in which exosome signals are transferred to the recipient cell by a three-step mechanism that involves the interaction of receptors, membrane fusion, and endocytosis (65). In addition, exosome uptake was measured quantitatively by using fluorescent EGFP Renilla protein (66). The exosome uptakes through the following processes are studied widely: clathrin-mediated, caveolin-mediated, macropinocytosis, phagocytosis, involvement of lipid rafts, and membrane fusion (67–72).

### Exosome Loading

Numerous methods have been proposed for loading exosomes that are classified widely into two different strategies: cargo loading after isolation and cargo loading during formation (73, 74). Exosomes composed of various types of cargo molecules DNA, RNA, protein, miRNA and lipids (75). The exosomal cargo loading is an important phenomenon for circulation and cell-cell communication. Endosomal cargo loading is differs in various physiological and pathological conditions. The endosomal sorting complex required for transport (ESCRT) a pivotal roles in synthesis and cargo loading (76). ESCRT family of proteins such as Tsg101, Hrs, CHMP4, STAM1, VPS4, VTA1, nSMase 2, PLD. CD9 and ALIX are important roles in cargo loading (77–81). N-linked glycosylation directed the glycoprotein sorting in extracellular membrane vesicles (82). Lipids plays a crucial roles in exosome biogenesis includes Phosphotidylserine (PS), phosphotidylethanolamine (PE), phosphatidylcholaine (PC), phosphatidylinositals (PIs), phosphatidic acid (PA), cholesterol, ceramide and spingomyelins (83, 84).

### Exosome Classification

**Figure 2:**
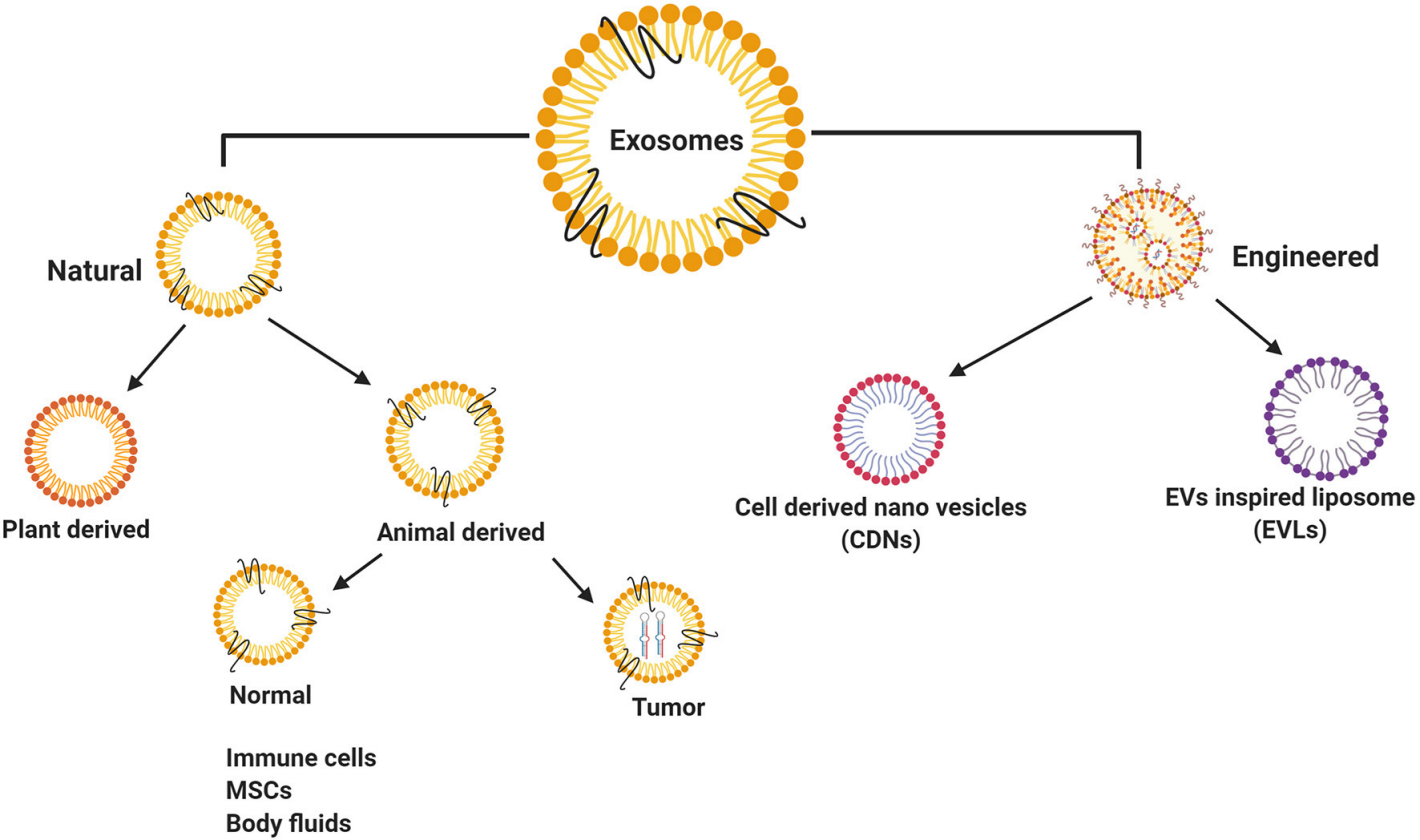
Classification of exosomes isolated from different resources. Natural exosomes have showed in animal/human derived exosomes, plant derived exosomes and engineered exosomes.

Exosomes have been classified broadly as natural exosomes (13) and engineered exosomes (85) (Figure 2). Naturally occurring exosomes are further classified into animal-derived exosomes (16) and plant-derived exosomes (86). As exosomes are synthesized under normal and diseased or tumor conditions, animal exosomes are subdivided further into normal exosomes and tumor exosomes or oncosomes (30, 87). Some of the normal cell type exosomes are derived from mesenchymal stem cells (MSCs) (88) and immune cells, such as T cells, B cells, macrophages dendritic cells, and natural killer cells (89). Among these, MSCs play a vital role in the disease development and are also involved in the damage and repair of tissues with definitive therapeutic prospects on CVD and certain neurological diseases (90). In addition, normal exosomes occur in body fluids, such as saliva, plasma, urine, milk, bile, ascites, and express diagnostic and therapeutic properties (13, 91, 92). Tumor exosomes are not only associated with tumor growth and metastasis, but also helps in identifying the disease conditions, thereby acting as ‘diagnostic markers’ (35, 93, 94).

## Biogenesis and Release of Exosomes

Initially, exosomes are synthesized with the inward budding of the cell membrane that gives rise to early endosomes processed by a secondary inward budding of the endosomal membrane to develop into numerous intraluminal vesicles (ILVs), also referred to as the late endosomes (13). Late endosomes enclosing ILVs are also known as multivesicular bodies (MVBs). In the end, the MVBs tend to fuse with the cell membrane bringing about the release of ILVs in an exocytotic manner outward into the extracellular environment (16). Originally, these ILVs are referred to as “Exosomes” (13, 80, 95–97).

Exosomes are released from the cell by either constitutive or inducible process. The constitutive secretory pathway is controlled by RAB GTPases (Rab 27a/b, Rab 11, and Rab 35), heterotrimeric G protein, WNT5A, glycosphingolipids, and flotillins (98–104). Furthermore, the inducible secretion pathway is controlled by stress stimuli that involve an aberrant intracellular calcium release, thrombin, DNA damage, hypoxia, lipopolysaccharides, and heat shock (99, 105–107).

Although the release is governed by either of these factors, ultimately exosome secretion necessitates its fusion with the plasma membrane (108). The membrane fusion of MVBs is facilitated by a combination of soluble N-ethylmaleimide sensitive factor attachment protein receptor (SNARE) proteins localized on MVBs that interact with target SNAREs localized on the intracellular side of the plasma membrane, which results in a membrane-binding SNARE complex paving pathway for membrane fusion (109–111).

A few pilot studies (112–115) have demonstrated that the exosomal RNAs are quite functional in their recipient cells by showcasing the composition of exosomes composed of double-stranded DNA (dsDNA), mRNA, and non-coding RNA. A study (81) depicts the efficient intercellular communication that arises due to the exchange of exosome contents across cells. In addition, evidence shows that the process of protein and RNA sorting into exosomes is dependent mostly on multiple pathophysiological factors, such as stress and disease (116, 117). This type of tailor-made exosomes with modified functional constraints due to any disease may serve as biomarkers for the diagnosis and prognosis of various diseases namely CVD (15, 20, 118).

## Stem Cells

Since the first study describing the potential of skeletal muscle in order to repair the heart, a wide spectrum of stem cells has been investigated for the treatment of CVD (119). Stem cells are capable of self-renewal with the ability to differentiate themselves into numerous cell types. Stem cells could be guided to become specific cells that can help to regenerate and repair diseased or damaged tissues (120). Stem cells are unipotent, multipotent, and pluripotent (121). Different types of stem cells exist and are classified based on their source, such as embryonic stem cells, umbilical cord blood stem cells, cardiac stem cells, MSCs, and so on (122).

The cell-based therapies were studied widely in cardiac tissue regeneration and the most significant type of cells identified are as follows: skeletal myoblasts (SKM) (123), bone marrow derived cells (BMCs) (124), induced pluripotent stem cells (iPSCs) (125), endothelial progenitor cells (EPCs) (126), and cardiac progenitor cells (CPCs) (127, 128). Among the three therapies identified, exosome-based therapy has emerged as the novel focal point to treat CVD (129). The various cell types that are studied widely in the case of cardiac repair *in vitro* and clinical trials are ESCs, iPSCs and multipotent/unipotent adult stem cell lineages, such as MSCs, cancer stem cells (CSCs), and cardiosphere-derived cells (CDCs) (130, 131).

Pluripotent stem cells are capable of differentiating into any type of cells in the body and used for tissue repair (132). Therefore, these stem cells were investigated widely as a harbinger of hope in cardiac regenerative therapy. To support this fact, Adamiak et al. (2) have stated that EVs derived from murine iPSC (miPSC–EVs) offer cytoprotective properties to cardiac cells *in vitro* and are capable of inducing superior cardiac repair *in vivo* associated with left ventricular function, vascularization, and amelioration of apoptosis and hypertrophy. A recent study (133) has revealed that the treatment using iPSC-derived exosomes induces the embryoid body differentiation and remodeled the cardiomyocyte hypertrophic cells.

### Stem Cell-Derived Exosomes

Pluripotent stem cells that are generated from adult somatic cells or blood cells by a process of genetic programming are called as iPSCs, wherein they revert into an embryonic-like pluripotent nature (134, 135). Owing to the clinical hurdles, such as safety and ethical issues, the use of human iPSCs for transplantation therapy to offer cardiac protection and restore function has been looked upon as a nightmare rather than a reality. Therefore, most of the research studies have been focused on secretomes (cell-secreted EVs) apart from iPSC injection/transplantation (133, 136). MSCs are the most magnificent producers of exosomes among the various cell types (90, 137, 138). The treatment of diseases based on the mechanism of MSC-derived exosomes has emerged as the future of scientific research. It has been put forth that therapeutic exosomes in tissue regeneration occupy the hotspot which might hold great promise for a long term.

In the 1970s, Friedenstein (47) described a population of bone marrow stromal cells that are capable of mesodermal differentiation and trophic support of hematopoiesis (48), Caplan (139) was the one who coined the term “Mesenchymal stem cells” in the early 1990s. MSCs constitute a heterogeneous subset of stromal regenerative cells and could be harvested easily from several types of adult tissues (140). MSCs have attained a prominent place by being known as the most promising of all therapy tools owing to their relatively simple procedure for cell isolation, self-renewal capacity, differentiation, ability, low immunogenicity, multipotency, and secretion of mediators that support tissue renovation or its substitution (140). MSC-derived exosomes seem to be enriched highly in certain biologically active molecules, such as RNAs and proteins (54). These MSCs are well equipped to maintain homeostasis within the tissue and respond to external stimuli. Studies have revealed that MSC-derived exosomes promote protective effects against myocardial I/R injury through several mechanisms that might include cardiac regeneration, anti-apoptosis, anti-inflammatory effects, cardiac remodeling, anti-vascular remodeling, and neovascularization (141–144). A recent discovery suggested that cardiac progenitor-derived exosomes inhibited cardiomyocyte apoptosis and improves the patients hailing in myocardial infarction and left ventricular ejection function (145).

As per a previous study (61), adipose-derived stem cells (ADSCs) are stromal vascular fragment of the adipose tissue in origin and have proved to be credible and reliable in terms of clinical implications. These cells have inherent differentiation properties that could give rise to diverse cell lineages and are capable of secreting high levels of protein that are known to play an important role in immunoregulation, revascularization, angiogenesis, coetaneous wound healing, and regeneration of worn-out tissues (146–148).

Apart from the secreted proteins, these cells are capable of releasing exosomes that are designated as small EVs with a multivesicular endosomal origin (13, 45). ADSC-Exos normoxic or hypoxic environment have been isolated successfully by using *in vitro* methods (149, 150). The work by Ribeiro-Rodrigues et al. (151) have demonstrated that cardiomyocyte-derived exosomes during ischemic condition gave rise to a new vessel formation and on further investigation revealed the proangiogenic effect, which was attributed partially to relatively expressed miR-143 and miR-222 in exosomes.

Another variant, known as a cardiac telocyte cell, that belong to the novel class of stromal cells was known to induce the growth and differentiation of CSCs/CPCs during organogenesis and brought about improvement in cardiac function (152). In addition, the transplantation of cardiac telocyte cells led to an enhancement of angiogenesis coupled with a decrease in cardiac fibrosis, which was involved in heart physiology and regeneration (153). Cardioprotective factors that enable beneficial effects to damaged heart were secreted by cardiac telocyte-derived exosomes (154).

A previous research work carried out by Kervadec et al. (155) stated that exosomes that are secreted by human ESC-derived cardiovascular progenitors (hESC-pg) were capable of providing equal benefits of cardioprotective effects in contrast to hESC-pg alone injected in a mouse with post-infarct heart failure (HF). Kervadec et al. (155) also identified over 927 upregulated genes in the heart owing to the treatment with hESC-pg and their derivative exosomes, which led to an increase in cardiac function by 78%. Khan et al. (156) have also observed that mouse ESC-derived exosomes help in restoring the cardiac function in acute myocardial infarction (MI)-infected mouse model. In addition, the study even reported that miR-294 was responsible for neovascularization, improved survival of cardiomyocytes, and reduced fibrosis post infarction.

Therapeutic efficacies of iPSC-derived exosomes have contributed to neovascularization and survival of cardiomyocytes in experimental animal models of CVDs, as demonstrated by Jung et al. (157). Besides these exosomes, iPSC-derived cardiovascular progenitor cells (iPSC-pg) have proven to be effective in the treatment of congenital cardiac failure (CCF), attributed mainly to the specificity of 16 highly abundant miRNAs that were known to be evolutionarily conserved mRNAs and involved in pathways associated with tissue repair (158). Khan et al. (156) have demonstrated that the mouse ESC-derived exosomes are capable of endogenous repair and preserve the functions of the heart when delivered by intramyocardial injection, as soon as left anterior descending ligation in an infraction-afflicted murine model. In particular, the researchers attributed the success toward ESC-derived exosomes, which were responsible for the beneficial effects in therapy to exosomal miR-294 (19, 156, 159). Cardiac progenitor-derived exosomes composed of pregnancy associated plasma protein A cleavage of insulin-like growth factor binding protein-4 and insulin-like growth factor-1 and activated the cell proliferation in Akt and ERK1/2 pathways in cardiomyocytes (160). In addition, cardiac progenitor-derived exosomes inhibited caspase activation and cardiomyocyte protection (160).

### Beneficial Role of Stem Cell-Derived Exosomes in CVD

Research data suggest that exosomes obtained from stem cells aid in cardioprotection, which promotes therapeutic remedy in the treatment of CVD (136, 161). Lai et al. (162) have demonstrated that human embryonic stem cells (hESC) secreted 50–100 nm membrane vesicles using an *ex vivo* Langendorff model of ischemia/reperfusion (I/R) injury, wherein they observed that these purified MSC-derived exosomes help in reducing the infarct size in mouse cardiac tissue.

**Figure 3:**
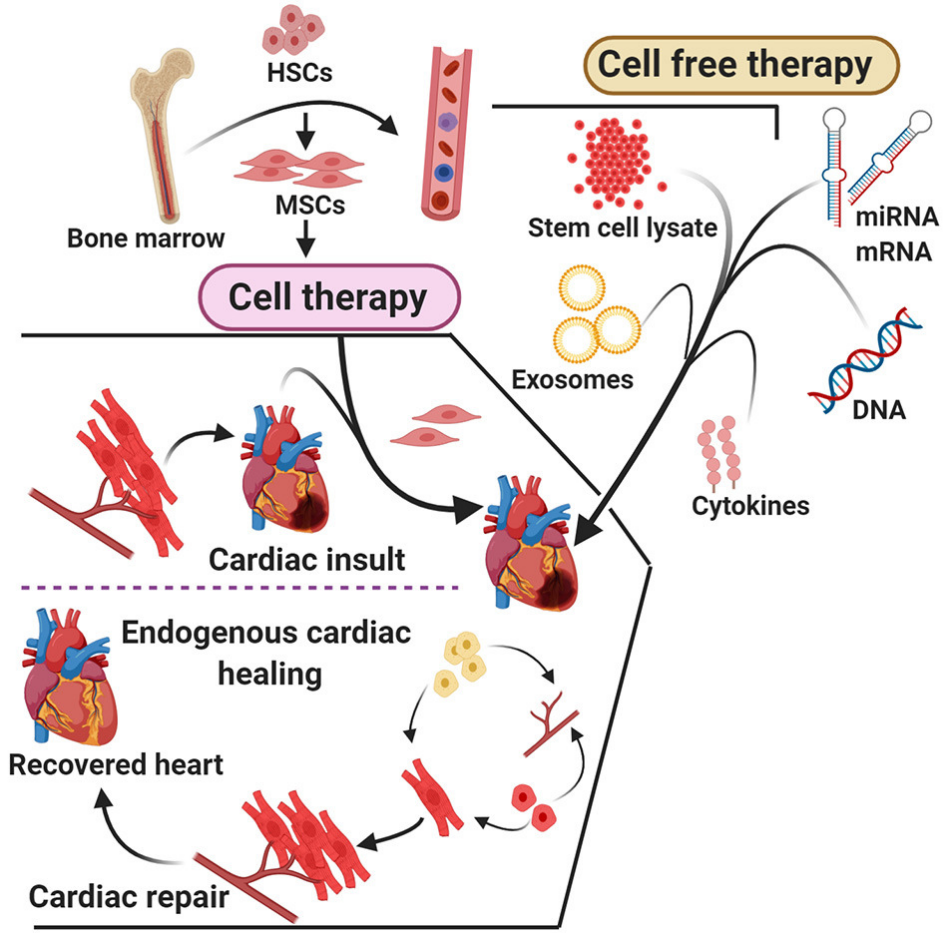
A head-to-head comparison of cell therapy and cell-free therapy.

Arslan et al. (163) showed that a single intravenous injection of exosomes (5 mm) before reperfusion reduced the size of infarct by ~45% in mice. It was also reported that the treatment with exosomes helped to restore depleted energy and redox rate potential in mouse health within 30 min after I/R, which was proven by the presence of increased levels of ATP and NADH levels with a decrease in oxidative stress (164). The studies conclude that MSC-derived exosomes harbor all necessary enzymes that are required for the generation of ATP (165, 166). The report also suggest that exosome treatment leads to the reduction in systematic inflammation in mice model after treatment with myocardial I/R (167) (Figure 3). Thus, MSC-derived exosomes could be a powerful therapeutic solution for patients diagnosed with acute myocardial infarction (167). The cardiac progenitor cells plays crucial role in angiogenic and cardiogenic properties in high-glucose conditions in both *in vitro* and animal models (168).

The data obtained by Wang et al. (169) revealed the supreme cardioprotective nature, inclusive of cell survival and angiogenesis by human endometrium-derived MSC (En-MSC). Mounting clinical and experimental evidences suggest the possibility that MSC with the ability to differentiate into different cell lineages could serve as a promising therapy to treat patients with cardiac dysfunction (170–172). Hematopoietic stem cell (HSC)-derived exosomes provide support to repair cardiac tissues by differentiating into cardiomyocytes (173, 174).

The research work published by Tseliou et al. (175) stated that the exosomes derived from cardiospheres (CDCs) seem to transform inert dermal fibroblasts into therapeutically active cells, which could bring about a decrease in the size of the scar and improve cardiac function in a chronic myocardial infarction model. CDCs are known to stimulate the regeneration of angiogenesis and functional improvement in the infarcted myocardium (176). Exosomes from CDCs could inhibit apoptosis significantly and are capable of promoting the proliferation of cardiomyocytes apart from enhancing angiogenesis (6).

### Adult Stem Cells (ASC) Based Therapy

Endothelial progenitor cells, a class of unipotent adult stem cells, have been looked upon as a therapeutic progenitor for the *post*-injury treatment of cardiac muscles (177, 178). A group of researchers (179) have demonstrated that adult human CD34^+^ stem cells isolated and purified from mobilized peripheral blood mononuclear cells (PBMCs) secrete exosomes (CD34 Exo) and are capable of inducing angiogenic activity in isolated endothelial cells and in murine models (179, 180). A study by Sahoo et al. (179) also reported a similar finding that human CD34^+^ stem cells have the ability to secrete cup-shaped exosomes that expressed CD63, phosphatidylserine, and TSG101. Recent striking discoveries shows that CD34-Exo are internalized easily by endothelial cells in the ischemic tissue, wherein they tend to induce cell cycle, angiogenesis, and cell proliferation (6, 181, 182).

On the basis of *in vivo* results, the scientists noted that both CD34^+^ cells and CD34^+^ exosomes induced the formation of vessel, such as endothelial cells, influenced by appropriately elevated proportion of endothelial cells in the Matrigel plug. A previous study (183) reveals that CD34^+^ exosomes form a key paracrine component of CD34^+^ cells induced vessel cell growth. Exosomes purified from body fluids and somatic cells have also been reported to be capable of being implemented as novel therapeutic options for certain CVD (36).

### Stroke and Stem Cell-Derived Exosomal Therapy

Stroke is a malfunction of the heart that leads to death and disability. Stroke is a form of degenerative disease associated with aging in individuals. Stem cell therapy could pave the way for a biological treatment alternative to the traditional therapy based on pharmacology (184). MSCs are chosen widely because of their differentiation ability and the ease with which they can be isolated from various adult tissues. In addition, adult MSCs pose less problems when compared with ESCs in terms of tumorigenesis and ethical issues (185, 186). In this regard, an evidence (139) claimed that the beneficial effects of stem cell transplantation are based on their ability to secrete bioactive molecules, namely, the exosomes. In a myocardial infarction model, the utilization of exosomes obtained from myc-transformed MSCs was known to reduce the infarction size (187). A previous study (138, 188) has focused mainly on the role of EVs in MSC transplantation and their therapeutic effects. The major subtypes of EVs are exosomes, microvesicles, and apoptotic bodies.

MSC-derived exosomes could be isolated steadily from MSC-conditioned medium. They are more effective compared with direct MSC transplantation used in the treatment of various ailments, such as CVD, acute kidney injury, liver injury, and lung injury (125, 185).

### Therapeutic Application of Exosomes

Exosomes contain various molecules, such as miRNA, that are capable of mediating biological function by means of gene regulation (185). miRNAs belong to a novel class of small non-coding RNAs (189, 190). Evidence show that miRNA are packaged into exosomes. They help to mediate cell–cell communication by acting as paracrine messengers (191). Exosomes serve as a new therapeutic target and could act as a biomarker for various disorders including cardiac disease (190). Mesenchymal stem cell derived exosomes (MSC-exo) used for various aging related diseases (192).

Exosomal miR-146a could be used as a highly specific blood biomarker that will help in the diagnosis and risk stratification of patients suffering from peripartum cardiomyopathy (115). Another studies (15, 193) has claims that the impairment of myocardial angiogenesis in diabetic patients occurs due to the secretion of anti-angiogenic exosomes released from cardiomyocytes. Sepsis-induced cardiomyopathy has been attributed to the high number of exosomes and their intrinsic properties (194).

Exosomes along with their biologically active cargos would offer apparent prognostic information about various diseases, such as chronic inflammation (195), cardiovascular and renal diseases (196), neurodegenerative diseases (197), lipid metabolic disorders (198), and tumors (199). Exosomes resemble the paracrine factors in both human and mouse CPCs (200) and stimulate heart protection inclusive of miRNAs both *in vitro* and *in vivo*. Gray et al. (201) reported that CPC-derived exosomes in response to hypoxia also led to the upregulation of 11 miRNAs compared with normal exosomes. In the previous work of Ong et al. (202), CPCs that overexpressed hypoxia inducible factor 1α (HIF-1α) help in improving the survival rate of transplants and the success was attributed mainly to the increased levels of miR126 and miR120 present within the exosomes, which had activated the prosurvival kinases and induced the glycolytic switch in the recipient CPCs.

Human CD34^+^ stem cell-secreted exosomes portrayed angiogenic activity, which was independent of its action in both *in vitro* and *in vivo* studies. This supports the notion that exosomes from stem cells could be an important component of therapeutic angiogenesis (11, 203). Ischemic heart disease (IHD) is caused by the blockage of blood flow to the heart arteries, resulting in reduced blood supply to the heart (204). It seems to be one of the most common cause of death, of over 9 million deaths per year worldwide (205). To cure this disease, stem cell therapy using somatic multipotent and pluripotent stem cells presents a valid approach to harness cardiac regeneration and myocardial function recovery (206). Giricz et al. (207) have demonstrated that EVs released from rat heart after ischemic preconditioning is essential and plays a crucial role in the transmission of remote conditioning signals for cardioprotection.

Exosomes deal with the transport and intracellular delivery of proteins and nucleic acids naturally. This helps us to rely on them as the attractive pharmaceutical delivery agents (208). Numerous commercial organizations have started developing exosome-based cancer diagnostics lately, such as Caris life sciences, exosomes diagnostics, and Humsa Bio Med. However, in the field of cardiovascular medicine exosomes remain an unexplored world that is committed to pioneer in order to reach the zenith of fruitful therapy.

Exosomes are known to offer atherosclerotic protein by means of cell–cell communication. Chen et al. (209) were able to trace the presence of RNAs in the supernatant of human MSCs that might be correlated to exosomes. They were able to witness a cardioprotective effect offered by these exosomes when infused into the rat model. Ibrahim et al. (210) have claimed the exosomes as critical agents that are capable of bringing about cardiac regeneration influenced by cardiosphere-mediated cell therapies. CPCs seem to possess antifibrotic effects by way of transferring the exosomes into fibroblasts therapy promoting angiogenesis and the survival of cardiac myocytes *in vitro* (85).

Exosomes that have been isolated from CDCs expressing Lamb 2b was known to contain cardiomyocyte-specific peptide on their surface and, therefore, led to the increased uptake by cardiomyocytes (85). Exosomes that were isolated from hiPSC-ECs contained miR-199b-5p and are known to promote angiogenesis (211). Exosomes have been synthesized naturally within the body and promote cell–cell communication, molecular therapy for cancer treatment (212), and in the diagnostics of various skeletal disorders, such as osteoarthritis (213), Osteochondral regeneration (214), myocardial I/R injury (162), limb ischemia, and pulmonary hypertension (215). Exosomes have been explored widely to act as the natural drug delivery vehicles, because they can travel safely in extracellular fluids trespassing immune cells and deliver the cargo to designated target cells with both utmost efficiency and a high degree of specificity (216, 217).

When it comes to exosomes as a notable therapeutic delivery system, it offers a few benefits in terms of specificity, safety, and stability. Exosomes are capable of delivering cargo to specific targets located at distant sites and could be employed to deliver small interfering RNA (siRNA) or other pharmaceutically active compounds (218). The miniature size of exosomes, being native to animals, helps them in avoiding phagocytosis easily, capable of fusing diligently with the cell membrane and escape the engulfing by lysosomes. As if the exosomes are a natural product of our body, which results in minimal or low immune response (219). In addition, exosomes possess a hydrophilic core that make them apt to deliver water-soluble drugs (220). ADSC-exosomes seem to play prominent tissue engineering and regenerative therapies (61). Exosomes have been used for therapeutic applications of cardiac ischemic diseases (221).

Transplantation of stem/progenitor cells have been declared as one of the promising therapeutic strategies for CVDs with the ability to replace lost cardiomyocytes and improve contractility (132, 222). Interestingly, exosomes have been stated to bring about communication among the cardiac fibroblasts, endothelial cells, and cardiomyocytes via delivering a wide array of contents inclusive of proteins and nucleic acids, such as RNA and DNA (108). This seems to be essential in order to support myocardium with oxygen and nutrients detrimental in normal heart and, thereby, mediate homeostasis of the heart maintaining structural integrity. In addition, exosome-enriched miRNAs nurture great promise for predicting the risk of developing CVD. Li et al. (55) have identified and listed plasma exosomal miR-422a and miR-125b2-3p, which might serve as a blood-based biomarker for the diagnosis and monitoring of patients suffering from ischemic stroke (IS). Recently, mesenchymal stem cell derived exosomes used in therapeutic purposes in SARS-CoV-2 infected cases (223). Recently, the exosomes which induces the autophagy and metastasis of tumor cells (224). Exciting results indicated that a long-term treatment of low-dose of Ticagcelor enhances the release of cardioprotective-exosomes through increasing cell proliferation in cardiomyocytes *in vitro* models (225).

### Advantages of Exosomes

Exosomes are smaller in size and being nanovesicles, they are less complex and less fragile than parent cells. They could be easily modulated/modified, engineered, manufactured, and stored. They evince no risk of tumor formation and are considered to be less immunogenic. They offer safety to their molecular cargos by providing the protection against enzymatic and non-enzymatic degradation (226).

First, the use of exosomes helps to avoid problems pertaining to cells that have defective or mutant DNA (227). Second, their small size makes it feasible to circularize easily through capillaries, whereas cells used in cell-based therapies, such as MSCs, might be large in size to traverse through the capillaries. Third, level of MSCs might diminish quickly after transplantation, whereas exosomes might be able to achieve a higher benefit than the transplanted MSCs (24). Fourth, exosomes lack toxicity and immunogenicity (228). It has been evident from various studies (229–232) that exosomes and their contents have the innate ability to govern cell survival, proliferation, migration, and differentiation in the damaged heart; thereby, reducing the risk of CVD-related deaths.

### Limitations/Disadvantages of Exosomes

It is really tough to note that a remarkable challenge lies ahead in developing scalable and reproducible protocols for isolation, purification, and storage of exosomes. In addition, there is a great deal of efforts necessary to provide improvised techniques and frame essential or vital norms for the analysis of the quality of exosomes. The suitable method to isolate sterile exosomes should be in compliance with good manufacturing practices capable of reproducible purity and potency (23). It is also difficult to set up a particular unit for determining the dose of exosomes, dosage regimen, particle numbers, and the route of administration (233). There is a great deal of effort and efficiency required to design a successful protocol to identify, quantify, and characterize the major exosomal component that are responsible for the biological effect in a particular disease. In addition, proposing the mechanism of action through qualified potency assays in case of relevant disease *in vitro* and in animal models is a tedious task requiring a lot of perfection with utmost efficiency (234).

### Future Perspectives

Exosomal research an emerging area of study which implies various advantages for therapeutic and diagnostic applications in cardiovascular disorder and other disease conditions. The future perspectives is need to address the route of administration of exosomes, dose fixation, biological half-life will be identified. The engineered exosomes are required to be tested in toxicity, immunogenicity and allergic potentials.

## Conclusion

Exosomes has a wide array of sources from stem cells to body fluids. Not only the regeneration potency of the exosomes, but also the cardioprotective roles of exosomes have been researched in many *in vivo* and *in vitro* findings. Exosomes are best studied for multi-targeted system biology for repair and regeneration as it harbors number of molecules. Based on the research purpose, the required therapeutic exosomes can be developed. To achieve this, a sensitive technique dissection and detailed analysis of exosomes components are required which will ultimately lead us to early diagnosis and standard treatment. This detailed review shows that the exosomes are effective and efficient drug delivery models. They also seem to be a promising path in the field of regenerative medicine. The stem cell-based therapy has emerged as the promising and safe therapeutic strategy for CVD. In conclusion, exosomes derived from stem cells is capable of exerting beneficial therapeutic effects on myocardial regeneration, thereby help in the treatment of CVD. In addition, clinical trials are required to prove the therapeutic efficacy of exosomes in CVD.

## Author Contributions

SJ and DG wrote the manuscript. JR and KP edited the manuscript. All authors contributed to the article and approved the submitted version.

## Conflict of Interest

The authors declare that the research was conducted in the absence of any commercial or financial relationships that could be construed as a potential conflict of interest.

## Publisher's Note

All claims expressed in this article are solely those of the authors and do not necessarily represent those of their affiliated organizations, or those of the publisher, the editors and the reviewers. Any product that may be evaluated in this article, or claim that may be made by its manufacturer, is not guaranteed or endorsed by the publisher.
